# Whispering sweet nothings: a review of verbal behaviors that undermine the effectiveness of government-mandated home-loan disclosures

**DOI:** 10.1186/s41235-019-0154-7

**Published:** 2019-02-13

**Authors:** Jessica M. Choplin, Debra Pogrund Stark

**Affiliations:** 1 Department of Psychology, DePaul University, 2219 North Kenmore Avenue, Chicago, IL 60614-3504 USA; 2 The John Marshall Law School, Chicago, USA

**Keywords:** Consumer protection, Persuasion, Disclosures, Home loans, Mortgages, Financial counseling

## Abstract

Home loans are the largest financial transaction consumers typically enter and the consequences from entering overpriced or unaffordable home loans can devastate individuals and entire communities. This article reviews how insights from research in experimental psychology can be utilized to protect consumers. Current policy relies too much on disclosures, which—among other limitations—can be undermined by verbal behaviors on the part of salespeople. In particular, salespeople such as mortgage brokers and lenders can exploit the fact that consumers do not know where to look for information on disclosure forms by violating conversational norms, introducing confirmation biases, and using dual tasks such as talking to consumers while they are reviewing forms. They can also exploit the fact that even consumers who know where to look can forget by part-set cuing consumers. They can even cause consumers who discover problematic terms to ignore them by providing the consumers with explanations. Policymakers need to be aware of these findings to design effective consumer-protection policies. The authors suggest alternatives for policymakers to consider.

## Significance

This article describes the implications of findings from research in cognitive and social psychology for consumer protection policy. The real-world implication of these findings is that psychological phenomena such as the influence of verbal information on visual search, conversational norms, confirmation biases, dual tasks, part-set cuing memory effects, and the effects of explanations on compliance undermine the effectiveness of disclosures to protect consumers. Research on these psychological phenomena suggests alternative policy prescriptions that policymakers should consider.

Home loans are the largest financial transaction consumers enter into (Bureau of Labor Statistics, 2017), and—due to the variety of features they can embody—they are also very complicated to parse (Choplin, Stark, & Mikels, 2013). Poor decision making when entering into such transactions can have dire consequences for the individuals who enter into them, including high rates of default and foreclosures (Federal Trade Commission, 2000). These dire consequences were seen most profoundly during the real estate recession starting in 2008 and its aftermath (Herron, 2013). Poor individual decisions on home loans can even have a strong negative impact on entire communities (Goodwin, 2016) and the general economy (Stiglitz, 2010). Due to the importance of making prudent home loan decisions and to protect consumers from overpriced and unaffordable home loans (two examples of “predatory home loans”), the federal government has increasingly regulated this industry (Dodd-Frank Wall Street Reform and Consumer Protection Act, 2010; henceforth Dodd-Frank; Real Estate Settlement Procedures Act, 1974, henceforth RESPA; Truth In Lending Act, 1968, henceforth TILA).

The federal government’s primary strategy in regulating this industry, however, has been a policy of mandating the disclosure of loan terms at the time the loan application is made and just before the funding of the loan. Prior to 2015, home loan consumers received the TILA and HUD-1 (Housing and Urban Development) RESPA forms to disclose the offered loan terms. Since 2015, they have received the Consumer Financial Protection Bureau’s (CFPB) Loan Estimate and Closing Disclosure forms. There are many problems with relying on disclosures alone to protect consumers. We have reviewed some of the cognitive and social psychological reasons why this regulatory approach is ineffective elsewhere, including problems such as consumers’ inability to process user-unfriendly features of disclosure forms, lack of contractual schemas, biases in assessing risk such as reliance on availability heuristics, reason-based decision making, biases in attribute evaluation and estimation, sunk cost and endowment effects, and temporal and uncertainty discounting (Stark & Choplin, 2009, 2010). This review article focuses on the related topic of how verbal behaviors on the part of salespeople such as mortgage brokers and lenders can undermine the effectiveness of disclosures, making the policy of relying on disclosures alone to protect consumers unwise.

The disclosure approach to consumer protection was first adopted during the late 1960s and early 1970s due to concerns that homeowners lacked the information necessary to make wise home loan decisions. Thus, Congress enacted TILA in 1968 and RESPA in 1974, which required lenders to disclose to consumers who apply for federally related home mortgage loans certain key economic loan terms. Congress presumed that borrowers would carefully read the disclosures, understand the basic terms of the proposed loan, and thereby be empowered to make wise decisions on whether to accept or reject an offered home loan. The assumption was that once consumers received this information, they would be able to shop for a loan that best suited their needs and goals. They would naturally select the best available loan for which they qualified; and since the assumption was that consumers understood the information presented in these disclosures, they would be granting their informed consent to the terms of any loan they entered. Requiring disclosure as a means to protect consumers from entering into problematic home loans was viewed as preferable to directly regulating the terms of these loans (such as requiring a maximum interest rate, maximum fees/costs, or imposing statutory maximum limits on the amount of mortgage debt based upon the borrower’s income) based upon the notion that under a free market with informed decision makers the parties can best maximize their utility and exercise their personal autonomy.

The policy of using disclosures failed to protect consumers for numerous reasons. Some of those reasons involved the cognitive and social psychology of consumers (the focus of this review), but there were also problematic changes in the industry. Over the following decades, home mortgage loans became increasingly complex and susceptible to abusive practices by lenders and mortgage brokers, which contributed to the mortgage crisis in the late 2000s (Carrozzo, 2005; Korngold & Goldstein, 2015; Woodward, 2003). Thus, disclosure forms proved not to be very helpful in adequately highlighting and informing many home loan consumers on the terms of their proposed loan, especially any problematic terms, and whether it would be wise to enter into it. For example, many consumers had difficulty identifying when loans had adjustable interest rates on the TILA and HUD-1 forms that were used prior to 2010 (Stark, Choplin, & LeBoeuf, 2013). Nor did these disclosure forms lead to consumers shopping for a better home loan as desired (Renuart & Thompson, 2008). In response, various arms of the federal government have tried to revise them to be more “user-friendly” and useful to borrowers. The Department of Housing and Urban Development created a revised HUD-1 RESPA disclosure form in 2008 that became effective in 2010 and which changed the form in order to, among other things, better highlight the adjustable rate features that a loan may have. After the great real estate recession, Congress created the Consumer Financial Protection Bureau to improve the disclosures further (Dodd-Frank, Section 1032[f]), and to outlaw some of the most harmful loan products (Dodd-Frank, Sections 1402 and 1403). But the policy emphasis continues to be that of mandatory home loan disclosures as the primary means to prevent home loan borrowers from entering into loans that are overpriced (higher in interest rates, fees and costs than they qualify for), unaffordable (based on their income), or risky (such as containing interest rates that can dramatically rise during the term of the loan). The Consumer Financial Protection Bureau (CFPB) produced its own home loan disclosure forms which have been in use since 2015. The CFPB forms combined two separate forms (the HUD-1 and the TILA) into one form when consumers receive an offer (Loan Estimate) and another form that consumers receive at closing (Closing Disclosure) and revised how the APR (annual percentage rate) is disclosed so that it is less conspicuous.

The effectiveness of disclosure forms has been the subject of some empirical testing. For example, the Federal Trade Commission (FTC) proposals for how to revise the TILA and HUD-1 RESPA disclosure forms were tested and consumers were found to answer more questions accurately after reviewing the FTC’s proposed disclosure form than after reviewing the TILA and HUD-1 disclosure forms (Lacko & Pappalardo, 2007). Likewise, the change in how the APR is disclosed under on the CFPB loan estimate form from the dual HUD-1 and TILA disclosures that were previously used was based on consumer testing reflecting that consumers were confused by APR and could not define it correctly (Kleimann Communication Group, Inc., 2009; Stark et al., 2013). This testing, however, was mostly concerned with the physical layout of the disclosure forms, the ability of consumers to comprehend what was being disclosed, and the ability of consumers to identify the lower cost loan when two different loans were presented in disclosure documents. The problems with disclosures are not limited to issues involving the physical layout of disclosure forms, but also include numerous issues in consumer psychology. Active research in judgment and decision making and related areas of cognitive and social psychology provides many insights into barriers that undermine the effectiveness of home loan disclosures (Barr, Mullainathan, & Shafir, 2008; Stark & Choplin, 2009, 2010; Willis, 2006). These fields of study can also provide insights into how verbal behaviors of the part of salespeople—their whispered sweet nothings—can undermine the effectiveness of the disclosure approach to consumer protection.

The primary psychological reason why disclosures are less effective than Congress originally hoped and also why salespeople’s sweet nothings can undermine their effectiveness is that the sheer volume of information consumers need to absorb is excessive (Barr et al., 2008). For example, in one case the court noted that it took the consumer two hours and forty-five minutes to read the car purchase and finance documents (*Castellana v. Conyers Toyota*, 1991). In another case, the court noted the problem of consumers being presented with “an incomprehensible number of additional forms to sign at closing” in a home loan transaction (*In re: T.V. Dukes*, 1982). The number of issues that mortgage consumers must consider and the complexity of those issues is inordinately large, such that home loan mortgage decisions are almost certainly among the most complex decisions that consumers will face in their lifetime (Choplin et al., 2013). There are cognitive limitations on what people can remember (Miller, 1956; Ratcliff, Clark, & Shiffrin, 1990; Roediger III, 1973) and consider when making decisions (Lussier & Olshavsky, 1979). Dual process models provide a valuable way to think about these limitations (e.g., Chaiken’s heuristic-systemic model, Chaiken & Ledgerwood, 2011; Kahneman, 2011; Petty and Cacioppo’s Elaboration Likelihood Model, Petty & Cacioppo, 1986). A number of researchers have even proposed that these models should serve as a general theoretical framework to think about consumers’ vulnerabilities to fraud and scams (Langenderfer & Shimp, 2001; Lea, Fischer, & Evans, 2009; Rusch, 1999; Whitty, 2013), which would include their vulnerabilities to predatory home loans.

Dual process models propose two different information-processing paths for making decisions (Petty, 1986). Under some situations, consumers are able to take the central processing route (Petty & Cacioppo, 1986; called System 2 by Kahneman, 2011) and carefully think through the options given all of the available information, but to do so they need to be highly motivated and able to process an extraordinary amount of information (Petty & Cacioppo, 1986). Mortgage consumers are highly motivated because the mortgage decision is one of the largest financial decisions that they will make in their lives. However, the decision-making process involves so many unfamiliar concepts (e.g., APR; Kleimann Communication Group, Inc., 2009; Renuart & Thompson, 2008), unknown contingencies (e.g., how long they may hold the loan, future interest rates, and the total cost of housing including estimating real estate taxes and home insurance and repair costs; Choplin et al., 2013), and relational complexity (e.g., deciding between a loan with a low interest rate and high fees or a loan with a high interest rate and low fees; Stark et al., 2013) that it is also among the most complex decisions that they will make in their lives. Thus, they will often be unable to use central, System 2 processing (Choplin et al., 2013).

When consumers are unable to process information as thoroughly as they need or as quickly as they need using System 2, they rely upon System 1, which utilizes shortcuts and heuristics to make decisions (Chaiken & Ledgerwood, 2011) and they engage in more superficial, peripheral processing (Kahneman, 2011; Petty & Cacioppo, 1986). Furthermore, predatory mortgage brokers and lenders can likely encourage their customers to use this System 1 processing by providing credibility cues and promises of homeownership as a reward, as research has found these factors to be very effective at invoking System 1 processing (Fischer, Lea, & Evans, 2013; Langenderfer & Shimp, 2001; Lea et al., 2009; Rusch, 1999; the effects of these credibility cues and rewards are well-established in other domains, but future research should investigate how they operate in the context of home loans). Thus, despite the advantages of using System 2 processing and despite having higher motivation to engage in more complex, systemic processing for mortgage decisions than most of the other decisions that they make, mortgage consumers will too often be using peripheral, System 1, processing to make home mortgage decisions.

Because consumers are too often using peripheral, System 1 processing even when making decisions as important as choosing a home mortgage, they rely on heuristics (Willis, 2006) and other decision-making shortcuts (Stark & Choplin, 2010; see Chaiken & Ledgerwood, 2011 and Kahneman, 2011 for theoretical approaches to this type of processing) and, thus, fail to use the information presented in the disclosure form well. A long line of research on persuasion has demonstrated that people are more likely to rely upon authority and other credibility cues as a shortcut when they lack the capacity to process information slowly and carefully (Kiesler & Mathog, 1968; Petty & Cacioppo, 1986). Such credibility cues might include whispered sweet nothings, such as statements expressing benevolence (that the salesperson is on the customer’s side) and statements expressing expertise early in their interaction (Arndt, Evans, Landry, Mady, & Pongpatipat, 2014) and these cues could cause them to take an authoritative salesperson’s word and fail to carefully scrutinize the disclosure form (this phenomenon is well-supported in other domains, but future research should test how it operates in the context of home loans). Likewise, consumers who recognize the mortgage lender perhaps from seeing or hearing their advertisements might use the recognition heuristic as a shortcut (Goldstein and Gigerenzer, 2002) and go with the lender they recognize and, thereby, fail to scrutinize the information presented on the disclosure form (this phenomenon is also well-established in other domains, but future research should test how it operates in the context of home loans).

Alternatively, consumers might look for a justification or reason to choose one option over another, rather than weighing all of the pros and cons of each option (Shafir, Simonson, & Tversky, 1993). This strategy might cause consumers to skim the disclosure form and seize upon one salient attribute, such as the monthly payment, at the expense of all other attributes in making choices, and to fail to read the remainder of the disclosure form (Willis, 2006). Salespeople can likely sway people toward using this type of decision-making by asking for a justification or by raising issues of justifiability (Briley, Morris, & Simonson, 2000) or by using puffery that can serve as a justification (Alba, Marmorstein, & Chattopadhyay, 1992; Simonson & Nowlis, 2000; like the phenomena described above, this phenomenon is well-established in other domains, but future research should also test how it operates in the context of home loans).

One way home loan consumers reduce the amount of information they need to consider is to adopt strategies to reduce the amount of reading or to guide their skimming of contracts and disclosure forms (LeBoeuf, Choplin, & Stark, 2016; Stark et al., 2013; Stark & Choplin, 2009). The sheer volume of information in the disclosure form and other home loan documents also creates a situation wherein non-expert borrowers need to be led through the documents that they are required to sign. Yet, the necessity of being led through these home loan contracts and forms can be problematic and make these borrowers vulnerable to deception as well as—in some unexpected circumstances—innocently causing the borrower to fail to spot or remember an important problematic loan term.

Particularly problematic is the fact that, currently, mortgage brokers and lenders typically lead consumers through disclosure forms. For example, they might point out on the disclosure forms the monthly payment amount and loan amount, but skip over the APR (the interest rate plus fees and most closing costs expressed as an annual percentage rate) if the APR figure is higher than what the borrower is expecting. After highlighting a few of the non-problematic disclosure terms, the mortgage broker or lender then moves on to the next set of loan documents rather than asking the borrower to carefully read over the entire disclosure form. Consumers may still feel that they have “read” the disclosure documents and loan documents because they have had the documents explained to them in this fashion even though they have not really read over the disclosure documents in their entirety as Congress intended.

In Stark and Choplin (2009), 72.7% of participants self-reported that they had read all of the terms of the home loan documents and 21.2% reported that they skimmed the loan documents (6.1% admitted that they did not read any of the loan documents). We are skeptical of the claim made by those 72.7% of participants that they really read all of the documents, however, as it took over three hours for a highly financially literate person to actually read each of the words in these documents (Stark & Choplin, 2009). Although some mortgage brokers and lenders induce borrowers to accept the disclosure documents without any explanations of these documents, most of the time the mortgage broker or lender makes some attempt at explaining these disclosure forms on some level to the consumers. This process can make consumers feel as if they have read the disclosure documents when they really have not done so in their entirety.

In the following sections, we describe three of the problems that consumers face in reviewing home loan disclosure documents and how these problems leave consumers vulnerable to misleading verbal behaviors—sweet nothings—on the part of salespeople when they are led through disclosure documents. These three problems are: (i) consumers do not know where to look when they review disclosure documents; (ii) even if consumers know where to look, they may have difficulties remembering to do so; and (iii) even if consumers discover problematic terms, the terms can often be explained away. Once we have reviewed these problems and the cognitive and social psychological factors that aggravate them, we will discuss possible alternative policy approaches beyond disclosures alone that policymakers should consider.

## Consumers do not know where to look when they review disclosure documents

The first problem consumer’s face when they review disclosure documents is that they do not know where to look for information on the disclosure forms, even if they know what the terms mean and they often do not. Ideally, consumers would read and understand all of the documents in their entirety, but there is simply too much information to process so consumers have to skim, and skimming leaves them vulnerable to factors that bias where they look (*In re: T.V. Dukes*). For example, some irresponsible mortgage brokers and lenders deceived their clients by purposefully diverting the attention of the consumer from problematic provisions in the disclosure form by instead pointing out other provisions that were not problematic and then directing the consumer to sign the disclosure document, if applicable, or moving on to the next document. The United States Court of Appeals for the 9th Circuit described how one lender employed this deceptive sales practice:

“Loan officers would employ a standardized sales presentation to persuade borrowers to take out loans with high interest rates and hidden high origination fees or “points” and other “junk” fees, of which the borrowers were largely unaware. The key to the fraud was that loan officers would point to the “amount financed” and represent it as the “loan amount,” disregarding other charges that increased the total amount borne by the borrowers. First Alliance trained its loan officers to follow a manual and script known as the “Track,” which was to be memorized verbatim by sales personnel and executed as taught. The track manual did not instruct loan officers to offer a specific lie to borrowers, but the elaborate and detailed sales presentation prescribed by the manual was unquestionably designed to obfuscate points, fees, interest rate, and the true principal amount of the loan. First Alliance’s loan officers were taught to present the state and federal disclosure documents in a misleading manner, and the presentation was so well performed that at least some borrowers had no idea they were being charged points and other fees and costs averaging 11 percent above the amount they thought they had agreed to. Loan officers were taught to deflect attention away from things that consumers might normally look at, and the loan sales presentation was conducted in such a way as to lead a consumer to disregard the high annual percentage rate (APR) when it was ultimately disclosed on the federally required Truth in Lending Statement.” (*In re: First Alliance Mortgage Co.*, 2006).

When Congress enacted the federal laws mandating disclosure of the key terms of home loans, it considered the APR (the interest rate plus fees and certain closing costs annualized over the term of the loan) to be the most important loan term, yet some irresponsible lenders were able to guide borrowers so that they would fail to notice this term in the TILA disclosure form by deflecting their attention away from the term. More sophisticated borrowers knew to look for the APR figure and were less susceptible to deflecting attention away from this term, but irresponsible lenders were still able to deceive even these relatively sophisticated borrowers by structuring loans with more hidden charges such as a “prepayment charge” (a charge, which can run into thousands of dollars, that the borrower is obligated to pay if the borrower pays off the principal balance of the loan prior to the maturity date of the loan). The lender or mortgage broker could then employ the same technique of diverting attention away from these fees by pointing out the favorable APR figure and deflecting attention away from the prepayment charge.

One factor that causes consumers, especially unsophisticated ones, to be deceived when they are led through disclosure documents by irresponsible mortgage brokers or lenders is that by discussing some items but leaving out other critical items, these lenders are violating a conversational norm that cognitive psychologists call “the Gricean norm of quantity” (Grice, 1975; see also McCornack, 1992, for an analysis of conversational norms and how violations of these norms are deceptive). The Gricean norm of quantity says that a speaker should include important information and exclude information that is not important, so items that are left out must not be important. Withholding important information in this way is usually deceptive (Burgoon & Qin, 2006). Since the Gricean norm of quantity is a conversational norm that all speakers are expected to use to guide communication, borrowers may be justified in assuming that lenders and mortgage brokers are following it. Yet irresponsible mortgage brokers and lenders sometimes violate this norm. As previously noted, if the APR was higher than what the borrower qualified for, they would emphasize other loan terms that were not problematic (such as the initial monthly payment amount) and omit the APR; conversely, if the APR figure was not problematic, they would emphasize that term and ignore any problematic terms such as prepayment charges. Responsible financial advisors would attempt to follow the Gricean norm of quantity and focus on all of the problematic terms, spending the most time on the most problematic terms and the least time on the least problematic. If a particular proposed loan contains numerous problematic terms, however, the borrower may still have difficulty recalling all of the problematic terms explained to them by the mortgage counselor.

We recently conducted an experiment in our laboratory to test the idea that the Gricean norm of quantity could be used to direct attention towards unproblematic terms and away from problematic ones. Participants (half community participants who were paid for their time and effort and half students who received class credit) were led through the 2010 HUD-1 disclosure form while their eye movements were tracked. The experimenter explained (that is, defined) four of eight loan attributes while pointing out those attributes on a blank disclosure form. The experimenter said nothing about the remaining items. The attributes that the experimenter defined were counterbalanced between participants to control for specific item effects. After the experimenter was done defining those items, participants rated the importance of all eight of the target loan attributes, including those that had not been defined. Next, participants looked over the disclosure forms for as long as they wanted on their own accord. They were advised that some questions would follow on whether the disclosed loan is a good loan and that they would be paid $1 for every correct answer. Participants’ eye movements were tracked as they did so and the amount of time that they spent looking at items that were pointed out and items that were not pointed out was measured. Despite the fact that participants did not rate the defined attributes more important than the undefined, participants were more likely to skip undefined attributes and they spent more time looking at the defined attributes (LeBoeuf et al., 2016).

Another factor that can cause consumers to be deceived when they are led through disclosure documents by irresponsible mortgage brokers or lenders is that consumers often try to reduce their reading load by skimming disclosure forms looking for information that confirms that what they were told was true. They often fail to look for information suggesting that what they were told was false. They do this, even though testing whether what they are told was false would often be a more productive test strategy. Testing whether a statement is true is called a confirmatory test strategy, while testing whether a statement is false is called a disconfirmatory test strategy. Consumers use confirmatory test strategies by default. Disconfirmatory test strategies are difficult for consumers even if they know that they ought to use them, and many consumers do not even know that they ought to use them.

The famous experiment within the field of cognitive psychology that established this phenomenon was conducted by Wason (1960). In that experiment, he tested whether people use confirmatory or disconfirmatory test strategies in a task wherein he gave his research participants a series of three numbers—the number 2, the number 4, and the number 6—and told them that this series followed a rule (Wason, 1960). The participants’ task was to generate additional series of three numbers and he would tell them whether or not their series followed the rule. The true rule was: any ascending series of numbers. Few participants, however, thought of this broad rule, and instead either assumed that the rule required the numbers to ascend by 2 or to ascend by equal increments. Most importantly, they tested this assumption by generating additional series that followed the rule they had in mind (doing so followed a confirmatory test strategy). They rarely generated series that did not follow the rule. That is, they rarely generated series that ascended by 1, 3, 7, or 53, ascended by uneven increments, or descended (to do so would be to follow a disconfirmatory test strategy). Finally, when participants thought that they knew the rule, Wason had them guess what the rule was.

Because they had used a confirmatory test strategy, only 6 out of 29 participants produced the correct rule on their first attempt. If instead they had used a disconfirmatory test strategy, they would have soon realized that the rule did not require the numbers to ascend by 2 or by equal increments, but few participants did so. Since Wason’s seminal work, many studies have confirmed—and almost no studies have disconfirmed—Wason’s observation that people use confirmatory test strategies to verify the veracity of almost every claim they hear (Bogan & Just, 2009; Klayman & Ha, 1987; Rabin & Schrag, 1999; Thaler, 1987; Wheeler & Arunachalam, 2008; Wood & Lynch Jr., 2002). People almost never use disconfirmatory test strategies as long as the claim does not contradict other entrenched beliefs. Confirmation biases can affect people’s search for information (Snyder & Swann Jr., 1978), their interpretation of information (Westen, Blagov, Harenski, Kilts, & Hamann, 2006), and their memory for information (Snyder & Cantor, 1979)*.*

Likewise, consumers use a confirmatory test strategy when they read or skim disclosure forms. They only look for information that confirms what they were told and fail to look for information that disconfirms it. For example, borrowers might fall prey to a predatory adjustable rate loan because the mortgage broker tells them that the interest rate will be at a given relatively low rate. Even if they are skeptical of the mortgage broker’s verbal representation, they will try to allay their concerns by looking for evidence in the disclosure documents that the interest rate will indeed be at the given low rate. They typically do not (and often cannot) think of the alternative that the given low rate is only an introductory rate and that it will change later. One of the authors (Prof. Stark) witnessed a law student go through such a cognitive process in her real estate transactions class. In her lecture, Prof. Stark had casually mentioned that the prevailing prime rate at that time was at least 5%. At the end of the lecture, a student approached her and claimed that she was about to receive a loan at 4%. The mortgage broker had told her that the rate was 4%, and she had read the mortgage contract—probably skimmed—and saw the interest rate was indeed 4%. She did not notice that the contract was for an adjustable rate mortgage. Housing Action Illinois (2007) reported that the overwhelming majority of the borrowers they spoke with that were presented with a floating rate loan were surprised to find out from the counseling agency that the loan they were about to enter was an adjustable rate loan which could lead to a doubling of the interest rate within a few years of entering into the loan.

Inspired by this event and observations, we conducted several experiments designed to investigate the effects of confirmation biases on how consumers review disclosure documents (Stark et al., 2013). As was the previous experiment on conversational norms, this experiment was run using an eye tracker to monitor participants’ eye movements. The experimenter told half of the participants only the initial interest rate (but not the adjusted interest rate) and told the other half of participants only the initial monthly payment (but not the adjusted monthly payment). Participants were then invited to look over the disclosure form and told that they would be asked questions about the form and that they would be paid $1 for every correct answer. We monitored their eye movements as they looked over the disclosure form.

In one experiment, we used the version of the HUD-1 disclosure form that was in use during the run up to the mortgage crisis. This form did not disclose adjustable rate loans well. There was one sentence in the TILA disclosure form identifying the loan as an adjustable rate loan in the middle of the page with a checkbox in front of it. Participants who received this older form often demonstrated a confirmatory pattern of eye fixations. They looked at the stated interest rate, the initial interest rate, but many failed to continue looking to see the sentence identifying the loan as an adjustable rate loan.

In a second experiment, we used the version of the HUD-1 created in 2008 that became effective in 2010 that was developed in response to the mortgage crisis. On page 3 of this form under “Loan Terms” the third box is labeled “Your initial interest rate is” and the fifth box is labeled “Can your interest rate rise?” and it identifies how high it can rise. Likewise, the fourth box is labeled “Your initial monthly amount owed for principle, interest, and any mortgage insurance is” and the seventh box is labeled “Even if you make payments on time, can your monthly amount owed for principal, interest, and mortgage insurance rise?” and identifies how high it can rise. This version of the HUD-1 was indeed better and many participants successfully continued to look and found the adjustable rate information.

This result indicates that improved disclosure forms can indeed be helpful. We then ran a third experiment, however, in which we used the better form, but engaged the participants in conversation while they reviewed the form—thereby creating a dual task—asking them about locations around the Chicago area to take an out-of-town guest. Dual task paradigms are often used in cognitive psychology to assess whether secondary tasks use the same cognitive resources and, thereby, interfere with tasks of interest (Pashler, 1994). Strayer and Johnston (2001), for example, found that cellular telephone conversations interfered with people’s driving abilities even when the cellular telephone was hands free. Consistent with previous literature on dual tasks, we found that when participants were engaged in our secondary task answering questions about Chicago, their performance declined significantly. It was, in fact, no better than with the form that was used during the run up to the mortgage crisis, even though the form was indeed a better form (Stark et al., 2013).

These results demonstrate why reliance on disclosure documents alone to protect consumers will inevitably be unsuccessful, except for very sophisticated home loan consumers. Verbal behaviors by salespeople—sweet nothings—such as violating conversational norms, introducing confirmation biases, and talking to consumers can direct consumers’ attention away from critical information as they review disclosure forms towards misleading information. Even extremely well-designed documents leave consumers vulnerable to such misleading sales strategies.

## Consumers have difficulties remembering where to look

Even if consumers know what to look for and they are financially literate enough to understand what terms mean, another reason why disclosure documents alone are insufficient to protect them relates to the fallibilities of human memory. Consumers will often fail to remember where they are supposed to look for critical information on home-loan disclosure forms (for research on this issue in the domain of webpage design, see McCarthy, Sasse, & Riegelsberger, 2004; Murano & Sander, 2016). Even consumers who know that they ought to look at key loan terms can forget to do so or forget where on the form the critical information was presented. This weakness in human memory leaves a vulnerability that unscrupulous salespeople can exploit by whispering a sweet nothing. In particular, salespeople appearing to be helpful can remind consumers that they should look for certain types of information. These reminders will create part-set cueing effects.

Research on human memory has demonstrated that activating or recalling some of these items in a set perhaps as a cue to supposedly aid recall of the remaining items counterintuitively makes it more difficult to recall the other items. Roediger III (1973) demonstrated this phenomenon by giving his participants categories of items to be memorized (for example, birds: stork, robin, thrush, canary, parrot, egret, and wren). Some of his participants were later asked to recall the names of the birds that they were asked to memorize. The other participants were given some of the birds (for example, stork, robin, thrush) as a memory cue and then asked to recall the remaining birds. Participants who were given some of the bird names had more difficulty remembering the remaining bird names than did the participants who were asked to recall the entire list (see Pei & Tuttle, 1999, for a discussion of how this phenomenon affects hypothesis testing among professional auditors). Roediger explained this phenomenon by hypothesizing that activating some of the bird names created an inhibitory effect on people’s ability to activate the remaining names.

A similar phenomenon may happen when financial advisors inadvertently remind consumers to look at some terms, but not others. Consumers might know, for example, that they should check the APR, the interest rate, and the monthly payments. They might know that they should look to make sure that a loan does not have an adjustable interest rate or adjustable monthly payments or prepayment charges. If, however, a financial advisor inadvertently reminds the consumer to look at a few of these, the consumer may then have difficulty remembering to look at the remaining items. This phenomenon would not only affect unsophisticated consumers, but would also potentially affect relatively sophisticated consumers who know which loan terms they are supposed to check.

We ran a series of experiments investigating this phenomenon (LeBoeuf, 2014). In particular, we wanted to know whether reminding people to look at some loan terms causes them to forget to check others that they previously knew that they were supposed to check. There were several stages in these experiments. In the first stage, participants were given eight loan terms that they were supposed to remember to review. The experimenter took his time reviewing and describing all of the loan terms the participant was to review. Participants then viewed a 10-min sports video, as usually there is some delay between when consumers learn that they should review terms on disclosure documents and when they actually do so, and to capture the fact that many lenders and mortgage brokers spend time at closing talking about irrelevant topics. After viewing the sports video, participants reviewed the 2010 HUD-1 disclosure form. Before they started doing so, however, the experimenter off-handedly reminded half of the participants of some of the terms. The other half of the participants proceeded directly to review the form.

Participants were told to look over the form for as long as they liked and were advised that they would be asked questions about the loan and would receive $1 for questions that they answered correctly. They were also told that they would be asked to judge whether the disclosed loan was a good loan. Their eye movements were tracked as they reviewed the form. We found that participants who were reminded to look at a few of the loan terms were more likely, than those who proceeded directly to review the form, to fail to look at the remaining terms. Furthermore, of those that they did look at, they spent less time looking at them (LeBoeuf, 2014). These results demonstrate that consumers are vulnerable to part-set cueing—a whispered sweet nothing—when they are reviewing a home loan disclosure form. This finding provides another demonstration of why disclosure documents alone are unlikely to be sufficient to protect consumers.

## Problematic loan terms can often be explained away

Finally, even if consumers succeed in discovering problematic terms, unscrupulous salespeople can employ another strategy—a whispered sweet nothing—to get consumers to ignore those terms by talking nonsense or pseudo-profound “Bμ££$#*+” (Barr, Pennycook, Cheyne, Koehler, & Fugelsang, 2015, this academic work uses the actual slang English profanity, without the symbols that we use here, as a technical term of art) or by providing explanations (Langer, Blank, & Chanowitz, 1978). Offered explanations often do not need to actually explain anything to convince people. Eriksson (2012) found that participants who held post graduate degrees in math, science, or technology did not rate an abstract of higher quality when a math formula was added, but those who held postgraduate degrees in other degree fields did. Likewise, adding irrelevant neuroscience details to psychological explanations makes those explanations more convincing (Weisberg et al., 2008). Neuroscience details seem to add a reductive allure even when those details add no substance to explanations (Hopkins, Weisberg, et al., 2015). If restatements are dissimilar enough, circular explanations can be difficult for people to catch (Rips, 2002). The power of explanations for gaining compliance was demonstrated in a classic social psychology experiment in which the experimenter asked to cut in line to make copies at a photocopy machine (Langer et al., 1978). When the experimenter simply asked to cut in line, few allowed it. However, when the experimenter asked to cut in line “because I am in a rush,” many people allowed it. Furthermore, the explanation did not even have to make any sense. When the experimenter asked to cut in line “because I have to make some copies,” many people again allowed it. In this third condition, no new information was presented beyond what was known in the first condition in which the experimenter simply asked to cut in line. In that condition, too, the person making copies knew that the person making the request needed to make copies. A real, authentic explanation, then, is not necessary. Merely offering up verbiage that takes the syntax of an explanation is often sufficient.

This phenomenon applies to many interactions between consumers and salespeople. In one case in Highland Park, Illinois in the 1930s, for example, a potential buyer was considering purchasing a property, but at the time there was no transportation to the property (*Ginsburg v. Bartlett*, 1931). The real estate broker told the potential purchaser that a railroad line was being built from Chicago out to the town and that there would then be transportation. The purchaser later reviewed the purchase agreement and discovered that it had a clause in it stating that no representations had been made regarding a railroad line being built. The purchaser was confused by this and asked the real estate broker about it, but the real estate agent was able to explain it away telling her that it was just an old form. She proceeded to close on the property.

To explore consumers’ vulnerability to explanations like this even when they succeed in reading and objecting to problematic provisions, we ran several experiments in our laboratory (Choplin, Stark, & Ahmad, 2011). In one study, we found that 86.7% of participants who discovered that a consent form contained a term contrary to what had originally been promised—and objected—signed it anyway if they were told a false but plausible explanation for the presence of that term (“that is just an old form”), and were assured it would not apply; and 80% signed if presented with a senseless explanation (“that is just the way the form was drafted”). In a follow-up study, we found that the contrary term could still be explained away by a senseless explanation even when participants initialed that term. In that study, 61.5% initialed the problematic provision and signed the form once they were told that the problematic provision was just drafted that way. This sweet nothing—providing an explanation, even a senseless explanation—can cause many consumers to ignore problematic terms even after discovering problematic terms. This phenomenon provides yet another reason why disclosure documents alone are unlikely to be sufficient to protect consumers.

## Implications for policy

The cognitive and social psychological factors described in this article, along with many others (Stark & Choplin, 2010), make it unlikely that merely presenting disclosure documents alone to consumers will be sufficient to enable consumers to make wise home loan decisions and to protect them from predatory lending, no matter how “user-friendly” those documents are made (Choplin et al., 2013; Stark & Choplin, 2009). Indeed, even if a home loan disclosure form were created to try to contain all of the information and explanations that a consumer would need, such a form would lead to information overload, and the manner in which the consumer is guided through the disclosure form—sweet nothings—will affect what they learn from the form (Stark et al., 2013). There are several possible strategies that policymakers may want to consider to overcome the limitations of disclosures and the misleading effects of whispered sweet nothings. Possibilities include improved presentation of the controversial, but we believe very useful, “APR” figure (with a price tag and statement “lower is better for you”), interactive online disclosures, disinterested financial counseling from a trained home loan counselor, and updated enhanced anti-usury laws.

### Improved APR disclosure with price tag and “lower is better for you”

In Stark, Choplin, LeBoeuf, and Pizor (2014), we argued that APR is a critical feature that consumers need to compare loan costs, because it is the only term that combines both interest rate and most fees. Of note, in one study reported in that paper we found that without APR information only 44% of participants correctly identified which of two loans was less expensive, which was at chance level, but 77% were able to do so with an “Enhanced APR Disclosure” which presented the APR in a price tag icon identifying the APR as the feature that reflected the cost of the loan and noted that “lower is better for you” as not all consumers know that lower APRs are better. Despite the utility of APR for identifying lower priced loans (Renuart & Thompson, 2008), when the CFPB created its Loan Estimate and Closing Disclosure forms, the disclosure of the APR was moved from the top of the TILA disclosure form to the third page of the Loan Estimate form and the fifth page of the Closing Disclosure form. This change was made due to a number of criticisms of APR. One criticism of APR is that consumers do not understand it, but in Stark, Choplin, LeBoeuf, and Pizor (2014) we found that our participants were nevertheless able to use APR information even without understanding it once they were informed that the APR reflected the price of the loan presented in a price tag icon and were informed that lower was better. Many of the remaining criticisms of APR can be addressed using interactive disclosures.

Another criticism is that the APR is not helpful for comparing adjustable-rate to fixed-rate loans. The CFPB’s “Explore interest rates” tool (https://www.consumerfinance.gov/owning-a-home/explore-rates/) already addresses this problem by only presenting other fixed-rate loans when one is considering a fixed-rate loan and only presenting adjustable-rate loans when one is considering an adjustable-rate loan. Thus, if the CFPB were to create a similar tool for APRs, this concern about APRs would already be addressed. Yet another criticism is that the APR is inaccurate for consumers who plan to sell or refinance as APR is now calculated for the entire length of the loan. Interactive disclosures could query consumers to help them estimate how long they will carry the loan. The APR of the loan the consumer is considering could then be calculated based upon this duration as well as other possible durations under a variety of contingencies under the assumption that the consumer makes a balloon payment at the end of this period after selling the property or refinancing. Thus, interactive online disclosures could address many of the shortcomings of using APR to compare how expensive loan offers are.

### Interactive online disclosures

Short of individualized in-person counseling (discussed next), which opponents might argue is too expensive (Stark & Choplin, 2010), similar benefits might be possible with well-designed interactive online disclosures and these benefits could go well beyond addressing the shortcomings of APR discussed in the previous section (Stark, Choplin, LeBoeuf, & Pizor, 2014). Such disclosures could present information serially so that consumers will be required to look at critical information serially rather than all at once, as is currently the case when consumers receive paper-based disclosure forms. Such disclosures could also generate personalized norm information, such as information about interest rate offers received by local consumers with the consumer’s same credit score. As noted above, the CFPB already has an online tool that does something like this asking the consumer for information such as their location and credit score and providing a histogram of the interest rates that other consumers with their credit score and in their location have recently received (“Explore interest rates”; https://www.consumerfinance.gov/owning-a-home/explore-rates/). As noted earlier, we believe in addition to this type of information, the CFPB should include a similar tool for APRs. The online tool returns norm information: the median interest rate (Helson, 1964) and a frequency chart of common interest rates for consumers in the same situation, thereby providing range and frequency information (Parducci, 1965, 1995), similar tools could generate norms for other loan features. The importance of this type of information is striking as it makes otherwise incomprehensible numbers evaluable (Hsee, 1996). This strategy is also more likely than other interventions to encourage consumers to comparison shop as it resembles social norm interventions that have already been found effective in improving health behaviors (e.g., reducing binge drinking; Perkins, 2003) and conservation (e.g., reducing energy, Schultz, Nolan, Cialdini, Goldstein, & Griskevicius, 2007, and hotel towel use, Schultz, Khazian, & Zaleski, 2008) by educating consumers about others’ behaviors (i.e., alcohol consumption, energy use, towel reuse rates). Consumers see these norms and change their behaviors to conform to them. Home-loan consumers may similarly be motivated to reject loan offers that contain “predatory features” (such as being overpriced, unaffordable, or containing risky terms that make default more likely) when they see norms that differ from the problematic loan being offered (Stark, Choplin, LeBoeuf, & Pizor, 2014).

These interactive disclosures could also be programmed to identify problematic terms, highlight them, and require consumers to click on those highlighted problematic terms which would link them to information on why that term is problematic. The consumer might then be required to respond to that information presented in those links to verify that they did understand that term and were granting informed consent to that term before they can sign their acceptance of the offered loan. In addition to providing personalized norms that are necessary to empower rational consumer decision-making, these disclosures will be structured such that consumers would not be able to accept an offered loan without first seeing and learning about problematic loan terms (Stark, Choplin, LeBoeuf, & Pizor, 2014), thereby confirming that the consumer has granted informed consent to the loan terms. To avoid sunk cost and endowment effects, these online disclosures could also be designed to come in two stages as Stark and Choplin (2010) proposed: One before consumers even start shopping so they can be educated and informed about market norms they need to know before shopping and another after consumers have found a loan so it can be compared to the loans that other consumers in their same situation have received (same credit score and location). If policymakers decide to pursue these interactive online disclosures, they will need to be very well designed. The design will need to be tested and the effectiveness of the interactive disclosures will need to be continuously monitored and researched.

### Informed consent procedures including disinterested financial counseling

To ensure that all consumers understand the loans they take out and are, thereby, in a position to grant informed consent to the loan agreement, Congress might require all home-loan borrowers to go through “informed consent procedures” to maximize the likelihood that consumers understand the loan terms well enough to consent. Currently, older consumers who are eligible to take out reverse mortgages and wish to do so must receive disinterested financial counseling (from a trained, reverse mortgage loan counselor authorized to provide this counseling by HUD) to do so (Stark, Choplin, Mikels, & McDonnell, 2014). Congress put this policy in place because reverse mortgage consumers did not understand the loans that they were taking out (Section 255 of the National Housing Act; 12 U.S.C. 1715z-20) and were, therefore, not in a position to grant informed consent to the terms of the loan. Policymakers might consider providing similar financial counseling services to consumers considering conventional mortgages to ensure that they too are in a position to grant informed consent to their conventional loans. This initiative should be aimed at providing individualized information from a disinterested party who would have no financial interest in misleading them. The disinterested party would not be in a position to financially gain from misleading or false initial information that would lead consumers to seek to confirm that information. They would have no financial incentive to engage in distracting conversations with consumers, so while they will likely engage in small talk with these consumers they will also respect social norms regarding when others need to concentrate on a task. The social dynamic here is the same as when drivers can engage in conversation with passengers riding in the car who can see hazards—but not conversation over a cellular telephone—because the passengers inside the car can respect norms regarding when to be quiet Strayer & Johnston, 2001. The disinterested party would not be in a position to financially gain from violations of conversational norms or part-set cuing that would steer consumers away from critical information on the disclosure form or from senseless explanations. Because the disinterested financial advisors would act as people usually do when they are not otherwise incentivized (i.e., they would follow conventional conversational norms and other social norms), the problems outlined in this article would thereby be substantially reduced.

All home-loan borrowers would be required to go through these “informed consent procedures”. The informed consent procedures might start with a financial knowledge assessment. The assessment would consist of a series of questions designed to gauge consumers’ understanding of real estate and financial terms and to assess their ability to use disclosure forms to effectively evaluate an offered loan. We recommend either HUD or the CFPB create the assessment, and that the assessment should be available on an agency-controlled web site. Sophisticated consumers who do well on this assessment could opt out of receiving disinterested counseling on the grounds that they are already knowledgeable enough to grant informed consent, thereby making this additional requirement to take out a loan less burdensome for these borrowers. Having such an assessment will also ensure that counseling is provided to those who need it most. It might seem heavy handed to require all consumers who wish to take out home mortgages to go through these “informed consent procedures” that include at a minimum a financial knowledge assessment and possibly also counseling. However, short of such measures it is not clear that consumers who take out loans understand them and have granted informed consent to the loan terms. Indeed, the process of taking the assessment can be a form of educating the consumer on what are the key loan terms to be reviewing when deciding whether to take the offered loan and explain what those terms mean and how to determine if the terms offered are fair or not, and when not fair as applied to them, the importance of shopping around for a loan with better terms. The mortgage counseling will also benefit legitimate, non-predatory lenders as well, because only knowledge consumers who understand their loan terms are in a position to affirm that they intend to comply with their contractual obligations under the loan. Thus, it can lead to better consumer decision-making, and in doing so, reduce the exposure of mortgage lenders and brokers to claims of inducing consumers to take out unsuitable predatory loans. With better home loan decision-making, there should be fewer borrowers defaulting on home loans and this should help restore the reputation of the mortgage lending industry and the value of the mortgages in a future secondary mortgage market.

Under the counseling we propose, consumers who are not sophisticated enough to pass the financial knowledge assessment would receive an explanation of the terms of the loan for which they have applied. Because of the problem of information overload, consumers would also receive a determination from an independent, specially trained mortgage counselor about whether the loan appears to be: (i) overpriced wherein the interest rate and fees exceed what the borrower could have qualified for); (ii) unaffordable under which the ratio of debt to income is unacceptably high; (iii) has risky features such as adjustable rates or balloon payments; or (iv) otherwise unsuitable (for example, because the refinancing is likely to result in a net economic loss to the borrower if the borrower is refinancing an existing debt). We also propose that counselors be required to make a simple recommendation to consumers whether or not the proposed loan appears to be the best the borrower can obtain in light of the factors described above.

When appropriate, counselors could also help consumers shop for loans with better terms by, for example, directing them to and completing the CFPB’s online interest rate comparison tool (https://www.consumerfinance.gov/owning-a-home/explore-rates/) with them which allows consumers to enter their credit score, state, the price of their home and their down payment, as well as the type of loan that they are receiving, fixed or adjustable, loan term, etc., and receive a histogram of the rates that other consumers under very similar circumstances to them are getting. Because the CFPB interest rate comparison tool does not include the impact of varying closing costs and fees on the overall costs of the loan, they should also look at the closing costs and fees and try to get data on closing costs for loans in the area where the home is located to compare against the closing costs in the offered loan. The counselor could also help consumers look up their credit scores and use those scores to look up the current market rate APRs for loans with the consumer’s credit score. This is helpful since the APR includes many of the closing costs by annualizing those costs into the APR price of the loan.

Other features of the counseling could include Stark and Choplin’s (2010) proposal for a two-stage counseling process to avoid sunk cost and endowment effects: A short online session before consumers even start looking at possible homes and shopping for loans and a later, more involved in-person session during which a disinterested financial counselor could analyze and explain the terms of the mortgage that the consumers had already found and were almost ready to sign. Further details on the mortgage counseling we recommend and its costs and benefits are detailed in Stark and Choplin (2010) and Stark et al. (2013). If policymakers decide to pursue such disinterested in-person financial advising, the design and effectiveness of the counseling will need to be monitored and researched throughout the implementation.

### Direct regulation of home mortgage loan terms

Another policy option is for Congress and the CFPB to directly regulate the substance of the loan terms to reduce the likelihood of harm from consumers taking out home loans that are contrary to their interests. Such regulations could, for example, set a maximum interest rate and maximum fees and costs. There are problems with this approach, however. First, it is not clear what the cap on interest rates should be set at. It could not be set to a fixed specific rate since prevailing fixed interest rates for “prime loans” (loans to the highest qualifying borrower) go up and down. In the past 50 years, they have gone from a high of 21.5% in December of 1980 to as low as 3.25% in the wake of the Great Real Estate Recession in December of 2008. They were even as low as 1.75% in December of 1947. Thus, any such cap would have to be set at a rate that is a specified percent over a bench mark that prime loan rates are based on (treasury yields) and would have to account for the varying credit scores of the borrowers who would not qualify for a prime loan and would need to pay a higher interest rate to account for the higher risk. This could lead to complicated calculations of what would be “usurious”. Similar concerns arise when trying to devise caps on fees and costs. If these caps were set too high, the caps would not provide as much protection as would learning how to shop or being helped to shop for the best loan terms possible. If these caps were set too low, these caps could make it difficult for lenders and other service providers to continue to provide their services.

Congress could also strengthen statutory maximum limits on the amount of mortgage debt borrowers can take on based upon their income. Congress has taken some measures to address the problem of unaffordable loans by seeking to cap the percentage of debt to income for each home loan borrower. For example, to qualify for an FHA loan the maximum qualifying ratios for borrowers in 2015 such that monthly housing payments should not exceed 31% of gross monthly income, and total debt burden should not exceed 43% of monthly income, but with some exceptions. For other federally insured home loans Dodd-Frank creates incentives to banks to offer loans that meet the 43% cap but does not require it. Study should be performed on the loans entered into since the passage of this incentive and see how well it is operating or if further reforms would be appropriate.

Dodd-Frank and CFPB regulations have already regulated some problematic home loan provisions such as “yield spread premiums” that incentivized mortgage brokers to induce consumers to take out loans with higher costs than they otherwise would have qualified for. Dodd-Frank also prohibits lenders from financing abusive forms of credit life insurance (15 U.S.C. § 1639c(d)) and it required independent real estate appraisals and prohibited mandatory arbitration clauses (15 U.S.C. § 1639c(e)). It also placed a cap on how long after a loan’s origination consumers could be required to pay prepayment charges (15 U.S.C. § 1639c(c)).

## Conclusions

Disclosure forms alone have failed to adequately protect borrowers from entering into overpriced and unaffordable home loans for a variety of cognitive and social psychological reasons, including problems such as consumers’ inability to process user-unfriendly features of disclosure forms, lack of contractual schemas, biases in assessing risk such as reliance on availability heuristics, reason-based decision making, biases in attribute evaluation and estimation, sunk cost and endowment effects, and temporal and uncertainty discounting (Stark & Choplin, 2010). This review expanded on previous reviews by covering the psychology of how unscrupulous salespeople can use subtle verbal behaviors—sweet nothings—to undermine the effectiveness of disclosures. Some of these sweet nothings include things sales people might say to encourage peripheral, System 1 processing and reliance on certain decision-making heuristics, the influence of verbal information on visual search such as violations of conversational norms and confirmation biases, using conversation as a secondary dual task to distract consumers, part-set cuing memory effects, and the effects of explanations on compliance. In light of these cognitive and social psychological phenomena, policymakers should consider supplementing disclosures with more robust efforts to empower consumers with the information they need to avoid problematic home loans, and the resulting devastation such loans can cause. Such efforts can also further the goal of consumers granting informed consent to the home loans they enter into. These empowering tools include: (1) revising disclosure forms to include an APR depicted as a price tag with “lower is better for you,” as a reform that has been demonstrated to work; (2) well-designed interactive online disclosures that could tailor information for a particular consumer’s situation, highlighting the key terms to identify if the loan is overpriced or contains other problematic terms and, if it does, to show how to negotiate or shop for a better loan; and (3) counseling on the specific offered home loan from disinterested, trained mortgage counselors similar to the counseling currently provided to reverse mortgage loan consumers these authors have proposed (Stark, Choplin, Mikels, & McDonnell, 2014). We believe that these empowering tools can help more consumers to make better home loan decisions. This in turn will make it more likely for consumers of home loans to avoid the calamities that can come from entering into ill-advised home loans.
